# Oligoprogression in NSCLC with Other Actionable Oncogenic Drivers Beyond EGFR and ALK: An Emerging Entity

**DOI:** 10.3390/ijms27062643

**Published:** 2026-03-13

**Authors:** Ilaria Mariangela Scaglione, Adele Bonato, Alessandra Dodi, Marco Sposito, Serena Eccher, Alice Avancini, Daniela Tregnago, Jessica Insolda, Michele Milella, Sara Pilotto, Lorenzo Belluomini

**Affiliations:** 1 Section of Innovation Biomedicine-Oncology Area, Department of Engineering for Innovation Medicine (DIMI), University of Verona School of Medicine and Verona University Hospital Trust, 37134 Verona, Italy; 2 Medicine and Surgery Department, University of Parma, 43124 Parma, Italy; 3 Medical Oncology Unit, University Hospital of Parma, 43126 Parma, Italy; 4 Department of Neurosciences, Biomedicine and Movement Sciences, University of Verona, 37134 Verona, Italy

**Keywords:** oligoprogressive disease, non-small cell lung cancer, local ablative treatment, less common actionable oncogenic drivers, targeted therapy

## Abstract

Oligoprogressive disease (OPD) in non-small-cell lung cancer (NSCLC) is a clinical entity with peculiar behavior and treatment. OPD patients, during systemic therapy, may receive local ablative treatment (LAT) with survival benefit. The importance of OPD and the role of LAT has been comprehensively assessed in the setting of EGFR mutant and ALK-rearranged NSCLC during tyrosine kinase inhibitor (TKI) treatment, but it is still almost unexplored in the context of NSCLC harboring actionable oncogenic drivers other than EGFR and ALK. The aim of our review is to collect and discuss the available data about standard treatment in this latter setting, with special consideration given to the role of LAT in case of OPD in systemic treatment. Through a comprehensive PubMed and ClinicalTrials.gov search, we identified the available data and ongoing clinical trials addressing these aims. To date, only limited evidence supports the use of LAT in OPD involving NSCLC driven by these molecular alterations, mainly deriving from case reports and retrospective series. This highlights an unmet clinical need that warrants systematic and multicentric data collection to generate more robust evidence.

## 1. Introduction

In 2025, lung cancer represented the leading cause of cancer-related mortality worldwide, accounting for nearly 2.5 times more deaths than colorectal cancer in the United States [1]. A paradigm shift in the management of advanced non-small-cell lung cancer (NSCLC) came with the identification of oncogenic driver alterations [2]. The consequent development of tyrosine kinase inhibitors (TKIs) led to significantly improvement in survival. However, acquired resistance to TKIs represents a major challenge to long-term TKI efficacy. In this context, oligoprogressive disease (OPD) in NSCLC represents a clinically relevant scenario and is defined as progression in one or a limited number of metastatic sites during the administration of systemic treatment, with maintained control and/or response of the primary lesion and other metastatic sites. The cut-off number of progressive sites needed to consider an oligoprogressive disease is not univocal, although most of the studies mention a limit of five progressing lesions. According to the available data, this phenomenon may be explained through tumor clonality based on preexisting or acquired genomic and microenvironmental features in each metastatic site, which may lead to variable responses to treatment [3,4].

After an extensive discussion about its optimal definition and treatment, this clinical entity is now recognized by most international guidelines. According to the European Society of Medical Oncology (ESMO) Metastatic Non-Small cell lung cancer guidelines, local treatment should be considered in patients who experience progression in one or a few sites during TKI therapy to increase their long-term progression-free survival (PFS) [5]. Local ablative therapy (LAT) defines a plethora of treatments, such as stereotactic radiotherapy (SBRT) and radiosurgery (SRS), surgery, and other image-guided ablative procedures, aimed at achieving definitive local control of a limited number of progressive lesions, while maintaining ongoing systemic therapy. In the setting of oligoprogressive oncogene-addicted NSCLC, LAT is recommended by the current clinical guidelines as a strategy to eradicate spatially confined resistant clones, prolong the clinical benefit of targeted therapy, and delay the need for a systemic treatment switch [3].

This review summarizes the current evidence of OPD biology, patient selection, and the role of local ablative therapies in prolonging the benefit of systemic treatment, especially in the context of *other* oncogene-addicted NSCLCs.

## 2. Major Molecular Targets in NSCLC

The role of LAT is particularly well established in the oncogene-addicted disease setting, especially for Epidermal Growth Factor Receptor (EGFR) mutant NSCLC. Although the oncogene-addicted disease demonstrated a significantly better PFS response to TKIs if compared with non-oncogene addicted disease, progression (and oligoprogression) inevitably occurs due to several resistance mechanisms. A retrospective analysis of 266 patients’ progression patterns during 4 years of EGFR tyrosine kinase inhibitor (TKI) therapy showed 37.6% of oligoprogression over 62.4% of systemic progression [6], highlighting the huge amount of patients who could benefit from a more “aggressive” approach, which allows them to carry on taking TKIs and to maintain the benefit obtained for the primary tumor and on other metastatic sites, reserving the switch to second-line therapy only in cases of worsening progression. Considering the incidence of OPD in this setting, a plethora of studies explored the role of LAT beyond progression in OPD sites. A retrospective study on 206 patients showed a median overall survival (OS) of 37.4 months when the OPD patients were treated with LAT, maintaining TKI therapy [7]. Another retrospective study included an OPD cohort comparing patients who switched to second-line chemotherapy (*n* = 25) to those who received treatment beyond progression plus LAT (*n* = 24), showing a significantly longer median OS (28.3 vs. 17.1 m, *p* = 0.011) and median Progression-Free Survival 2 (mPFS2) in the second group (13.9 vs. 9.2 m, *p* = 0.007) [8]. Similar findings were reported in another retrospective analysis of patients treated with afatinib or gefitinib, where high-dose RT combined with continued TKI therapy was associated with a significantly longer OS compared with TKI beyond progression alone or switching to another systemic therapy (37.3 vs. 20.1 vs. 15.1 months, respectively; *p* < 0.0001) [9]. Of note, central nervous system (CNS) progression is a crucial resistance pattern in EGFR TKIs. In preclinical models, a combination of systemic treatment and a local approach appeared to have a synergistic effect on disease control in this site, although potentially burdened by severe neurotoxicity [10].

From a translational perspective, the assessment of circulating tumor DNA (ctDNA) through liquid biopsy has emerged as a potential biomarker to stratify patients who may benefit from LAT, while continuing to take tyrosine kinase inhibitors (TKIs). In a study focusing on EGFR mutant oligoprogressive disease, ctDNA positivity at the time of progression was associated with worse outcomes, suggesting that circulating molecular burden may reflect the presence of systemically resistant clones, and thereby assist in therapeutic decision making [11]. These findings support the role of ctDNA analysis in identifying patients with biologically more widespread progression, among whom local treatment alone may be insufficient, and who may therefore benefit from treatment intensification or modification of systemic therapy. Nevertheless, significant limitations remain in the ability of ctDNA alone to consistently discriminate between oligometastatic and systemic progression. Despite these constraints, liquid biopsy represents a valuable bridge between radiological assessment and underlying molecular dynamics [12].

Notably, the therapeutic landscape for EGFR-mutated disease is rapidly evolving in light of the FLAURA2 and MARIPOSA trial results, which have resulted in the approval of two combination approaches: osimertinib with chemotherapy and Amivantamab with Lazertinib [13,14]. In this context, optimal management of oligoprogression will need to be reconsidered in the future.

Anaplastic Lymphoma Kinase (ALK) rearrangement occurs in approximately 5% of advanced NSCLC cases, and the initiation of a specific TKI dramatically improves clinical outcomes; nevertheless, progression still occurs in most of the patients. Local approaches have been evaluated to manage those patients who develop extracranial oligoprogression to ALK-TKIs, delaying the switch to chemotherapy or to second-line TKIs. Weickhardt et al. examined 15 ALK-rearranged patients with OPD on first-line crizotinib, showing an additional 6-month benefit with LAT and continuation of target therapy [15]. In another study, LAT of the oligoprogressive sites allowed them to continue crizotinib for 28 months, versus 10 months in patients not suitable for local treatment, with a 12-month local control rate of 86% [16]. CNS metastases represent a crucial issue in the ALK-rearranged disease, and even though second- and third-generation ALK inhibitors show stronger CNS activity compared to first-generation TKIs, this district remains a frequent site of progression [17]. In a pooled analysis of the PROFILE-1005 and PROFILE-1007 trials, among 34 patients who received crizotinib beyond isolated CNS progression, 27 received brain radiotherapy, with a post-progression treatment duration of 19.3 weeks [18].

Moreover, the data support a biological rationale of combining systemic TKI therapy and radiation therapy [19]. EGFR mutant cells show increased double strand break (DSB) repair, cell proliferation and apoptosis inhibition, inducing radio-resistance [20]. On the other hand, EGFR inhibitors suppress DNA repair system, increase apoptosis and induce G1-G2 arrest, avoiding tumor cell maintenance in the S-phase [21]. Additionally, TKI and RT combination induces poly (ADP-ribose) polymerase (PARP) cleavage, reducing its activity and further suppressing the DNA repair system [10].

Similarly, in ALK-rearranged NSCLC, crizotinib showed radio-sensitizing activities in preclinical models by inhibiting the ALK downstream effectors (AKT, STAT3, and ERK1/2) [22] with promotion of anti-proliferative, pro-apoptotic and antiangiogenic radiation-enhancing effects [23].

## 3. Other Actionable Drivers in NSCLC

In recent years, the discovery of oncogenic drivers EGFR, ALK and ROS1 and their specific inhibitors allowed 25–30% of patients with advanced NSCLC to receive a potentially more effective and less toxic therapy, with increased response rates and improved survival outcomes compared to the classical first-line chemotherapy [24].

**Figure 1:**
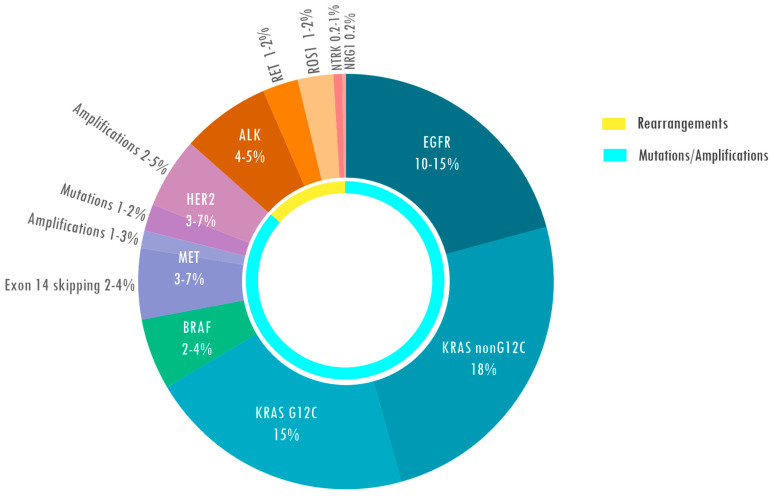
Molecular targetable alterations in non-small-cell lung cancer (NSCLC). EGFR, Epidermal Growth Factor Receptor; KRAS, Kirsten Rat Sarcoma; BRAF, V-Raf Murine Sarcoma Viral Oncogene Homolog B; MET, proto-oncogene receptor tyrosine kinase; HER2, Human Epidermal growth factor Receptor 2; ALK, Anaplastic Lymphoma Kinase; RET, REarranged during Transfection; ROS1, proto-oncogene tyrosine-protein kinase 1; NTRK, neurotrophic receptor tyrosine kinase; NRG1, Neuregulin 1.

The development and increasing availability in daily clinical practice of Next-Generation Sequencing (NGS) platforms highlighted several *other* molecular alterations in oncogenic driver genes and led to the development of specific drugs which showed impressive activity. Overall, it is estimated that about 60% of lung cancers are driven by molecular alterations, thus patients may potentially benefit from target therapy (Figure 1) [25].

Kirsten Rat Sarcoma (KRAS) mutations occur in approximately 30% of lung cancer cases. Its activity as an oncogenic driver was assessed 30 years ago, and it was considered as “undruggable” until 2019 when sotorasib and adagrasib, KRAS G12C inhibitors that irreversibly bind KRAS G12C in its GDP-bound state, showed an encouraging 40–50% objective response rate (ORR) [26,27], with an mPFS of 6 months. These results led to the Food and Drugs Administration (FDA) accelerating approval for patients with KRAS G12C-mutated NSCLC with at least 1 line of prior therapy for advanced disease. A recent analysis of 43 patients’ pre- and post-treatment specimens showed primary and acquired KRAS, NRAS and BRAF mutations as resistance mechanisms, suggesting a possible role of extracellular signal-regulated kinases (ERKs) signaling co-inhibition to prolong the benefit of a KRAS G12C blockade [28].

The BRAF (V-Raf Murine Sarcoma Viral Oncogene Homolog B) V600E mutation occurs in 2–4% of NSCLC cases, associated with a poor OS rate and a limited response to first-line chemotherapy. Based on the results of phase II trials showing a 63.2% ORR and an mPFS of 11 months, the dabrafenib–trametinib combination is FDA- and European Medical Agency (EMA)-approved for the treatment of these patients, regardless of prior therapy [29,30]. More recently, Encorafenib in combination with Binimetinib has been approved as a first-line therapy in this setting. In the PHAROS trial, a phase II trial, this regimen achieved a median progression-free survival duration of 30.2 months and an objective response rate of 75% among treatment-naïve patients [31]. Several resistance mechanisms to BRAF-V600E have been investigated, including onset of BRAF V660E, sustained ERK activation and Pi3K-Akt-mTOR pathway upregulation. Third-generation BRAF inhibitors and ERK1/2 inhibitors are currently under development to overcome these resistance mechanisms [32,33].

Advanced NSCLC may cause MET proto-oncogene receptor tyrosine kinase dysregulation both as a primary driver and as a resistance mechanism to EGFR TKIs. As a primary driver, MET amplification occurs in 2–5% of cases, while MET exon 14 skipping, caused by point mutations, insertions or deletions in the splicing site, represents 3–4% of patients [34]. Moreover, MET amplification occurs in the 10–20% of cases as a resistance mechanism to EGFR inhibitors [35]. Among the MET alterations, the exon 14 skipping mutation has the most solid evidence supporting its activity as a driver and thus encouraging its targeting with specific drugs [36]. In an expansion cohort of the PROFILE-1001 trial, the multi-kinase inhibitor crizotinib showed an ORR of 32%, with an mPFS of 7.3 months in patients harboring the exon 14 skipping mutation [37]. More recently, selective MET inhibitors such as capmatinib and tepotinib showed activity against the MET exon 14 skipping mutation in phase II trials with ORRs of 61% and 51%, respectively [38,39]. Interestingly, capmatinib showed also activity on brain metastases. Based on these results, the FDA granted the breakthrough drug designation as a first-line treatment for capmatinib and as a second-line treatment after first-line platinum-based chemotherapy for tepotinib. A third selective MET inhibitor, savolitinib, showed an ORR of 47.5%, with a Disease Control Rate (DCR) of 93.4% and median Duration of Response (mDoR) not reached in pre-treated patients with NSCLC with the MET exon 14 skipping mutation [40]. Resistance to MET inhibitors may arise with on-target or off-target mechanisms. In 2020, Recondo et al. published the results of an analysis on plasma or tissue biopsy data collected at the time of progression from 20 patients who developed resistance to MET inhibitors; 35% harbored new MET alterations, particularly new mutations on the kinase domain and amplifications, while 45% developed off-target resistance due to KRAS mutations and amplifications in KRAS, EGFR, Human epidermal growth factor receptor 3 (HER3), and BRAF [41]. The best strategy to overcome these mechanisms is still uncertain, as switching to another MET inhibitor or combining TKIs with other treatments to target resistance pathways may represent an effective option [42].

REarranged during Transfection (RET) rearrangements, involving at least 12 known fusion partners, occur in 1–2% of patients with NSCLC. These alterations are associated to less thymidylate synthase expression, which leads to a stronger response to pemetrexed [43]. Several multitarget agents (i.e., cabozantinib, vandetanib, lenvatinib, sunitinib, and nintedanib) showed some activity in NSCLC with RET rearrangements, but the ORR did not exceed 50%, and the toxicity profile was unfavorable [44]. In the ARROW trial, pralsetinib showed an ORR of 70% in treatment-naïve patients (including 11% of complete responses) and 61% in patients previously treated with platinum [45]. The LIBRETTO-431 trial showed mPFS durations of 24.8 months with selpercatinib and 11.2 months with the control treatment in treatment-naïve patients [46]. Both pralsetinib and selpercartinib demonstrated a manageable safety profile [47]. Bypass pathway activation, such as EGFR, MAPK, and MDM2, could represent one resistance mechanism to anti-RET drugs. Acquired MET and KRAS amplification have also been described [48,49,50]. The on-target mechanisms include the RETG810C/S and RETY806C/N non-gatekeeper mutations, which determine in vitro cross-resistance to pralsetinib and selpercatinib [51].

Human Epidermal growth factor Receptor 2 (HER2)-activating mutations have been observed in about 1.7% of lung adenocarcinomas, with the majority occurring in exon 20 [24], with HER2 amplifications and overexpression in 2% to 5% and 2% to 4% respectively [52]. In a phase II basket trial, trastuzumab emtansine (TDM1) showed a 44% ORR in pretreated HER2ex20 lung adenocarcinoma [53]. In the Destiny-Lung01 trial, trastuzumab deruxtecan showed a 55% ORR with a mDoR of 9.3 months and a mOS of 17.8 months [54]. Recently, in the Beamion LUNG-1 Cohort 2, zongertinib demonstrated substantial clinical activity in treatment-naïve patients with HER2 mutant NSCLC, with an ORR of 77% and disease control in 96% of cases, accompanied by durable responses (6-month DoR and PFS > 79%) [55]. Similarly, the SOHO-01 trial supports HER2-targeted TKI therapy with sevabertinib, showing an ORR of up to 71% in treatment-naïve patients and 64% in pretreated patients, with a median response duration of approximately 8.5–11 months [56]. Afatinib, a pan-HER inhibitor, demonstrated clinical activity in patients with rare HER2 mutations (i.e., V659E) [57]. NSCLC resistance to anti-HER2 therapy could develop a stem cell phenotype [57], MET amplification and upregulation of the Pi3k-Akt-mTOR pathway [24].

Neurotrophic receptor tyrosine kinase (NTRK) 1, 2 and 3 fusions are a rare group of gene alterations which occur in 1% of NSCLC cases. Recently, the FDA granted accelerated approval to two first-generation selective TRK inhibitors as tumor-agnostic drugs [24]. Larotrectinib, a panTRK inhibitor with strong CNS activity, has been investigated in three phase I/II clinical trials (NCT02122913, NCT02637687 and NCT02576431) involving patients with solid tumors harboring NTRK fusions. Among the patients with NSCLC, the ORR was 75% [58]. Entrectinib is a multitarget inhibitor, with activity on ALK, ROS1, TRK A/B/C and CNS, evaluated in three phase I/II trials; an integrated analysis from STARTRK-2, STARTRK-1 and ALKA 372-001 reported a 70% ORR in the NSCLC cohort, with an mDoR of 10 months and an mPFS of 11 months [59]. Resistance mechanisms to first-generation TRK inhibitors have been detected, including on-target mechanisms (mutations in the drug-binding site) and off-target alterations (MET amplification, BRAF V600E and KRAS mutation) [60,61]. Next-generation TRK inhibitors (repotrectinib and selitrectinib) will overcome these TRK on-target resistance mechanisms.

Proto-oncogene tyrosine-protein kinase ROS1 rearrangements account for 1–2% of NSCLC cases and are typically associated with a younger age, non-smoker status, and the female sex. The first-line options include crizotinib—first approved based on the PROFILE-1001 expansion cohort, which reported an ORR of 72%, a DCR of 90%, a median PFS of 19.2 months, and a median OS of 51.4 months [62]—and entrectinib. In the updated analysis by Dziadziusko et al. that included 161 patients with ROS1-positive NSCLC enrolled in STARTRK-2, STARTRK-1, and ALKA-372-001, entrectinib achieved an ORR of 68.1% and an mPFS of 15.7 months, but did not reach the median OS. Among the patients with brain metastases (34.8%), the intracranial ORR was 52.2% and the median intracranial PFS was 8.3 months, supporting entrectinib as the preferred option in this subgroup [63].

Approximately 50% of patients progress due to acquired ROS1 resistance mutations, particularly G2032R [64]. Repotrectinib, a next-generation ROS1/ALK/TRK inhibitor with activity against both the wild-type and G2032R-resistant variants, demonstrated tumor regression in most patients with G2032R mutations and showed high efficacy in the TRIDENT-1 trial (ORR 79% and mPFS 35.7 months in treatment-naïve patients) [65]. Based on these results, repotrectinib has received FDA-accelerated approval and EMA approval for both treatment-naïve and pretreated ROS1-positive NSCLC.

Other TKIs such as ceritinib and lorlatinib show activity in ROS1-rearranged NSCLC, though their impact on resistance mutations is less clear, while cabozantinib has demonstrated activity against G2032R and additional resistant variants [66].

## 4. Available Data and Future Perspectives on Oligoprogression in NSCLC Harboring *Other* Oncogenic Drivers

**Figure 2:**
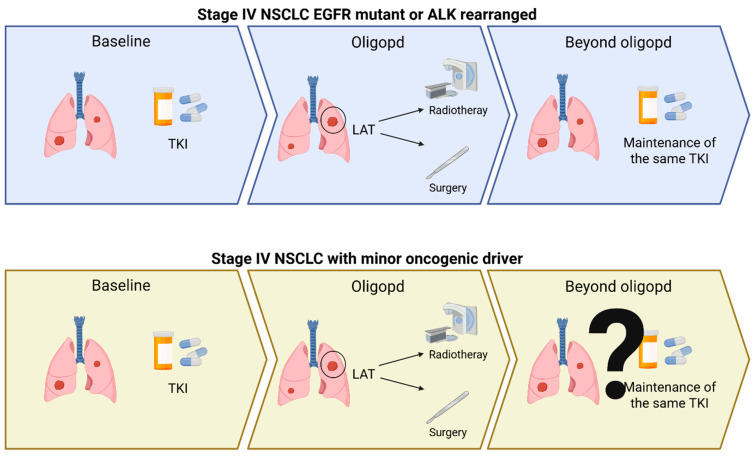
Therapeutic approaches to oligoprogression. NSCLC, non-small-cell lung cancer; Oligopd, oligoprogression; TKI, tyrosine kinase inhibitor; LAT, local ablative treatment.

Although specific drugs against emerging targets described above are increasingly used in daily clinical practice, only some data are available about oligoprogressive disease in this setting, suggesting an unmet need which may be investigated in future clinical trials, considering the astonishing ORR to target therapy of NSCLC patients harboring these alterations (Figure 2).

A recent retrospective study by Tsui et al., which included additional driver oncogenes such as ROS1, BRAF, and RET beyond EGFR and ALK, evaluated patients with ≤5 sites of OPD treated with newer-generation TKIs. The study demonstrated an overall PFS extension with LAT of 6.74 months and a global duration of treatment (calculated from TKI initiation to treatment switch) of 18.8 months. Although the number of ROS1+, BRAF+, and RET+ cases was small, the findings suggest that similar PFS benefits from LAT may be observed across different oncogenic drivers [67]. In general, most of the available data is represented by case reports. Therefore, a better characterization of oligoprogression in NSCLC harboring less common oncogenic drivers represents a current clinical need, particularly in light of the widespread adoption of comprehensive genomic profiling. In 2017, Occhipinti et al. reported the case of an 18-year-old non-smoking woman who received crizotinib plus whole brain radiotherapy (WBRT) for ROS1-rearranged NSCLC with brain metastases. After 7 months of treatment, intracranial progression occurred, and the patient underwent stereotactic radiosurgery of five progressing brain metastases, continuing crizotinib administration and maintaining a good performance status. The second intracranial time to progression (IT-TTP) occurred after 16 months, while a chest response was maintained for 23 months [68]. Wenzel et al. described a case report of a 75-year-old man with lung adenocarcinoma with bone and lymph node metastases harboring the BRAF V600E mutation. A first-line combination of dabrafenib-trametinib provided a partial response maintained for 18 months, and then a CT scan showed an increase in a single lesion in the contralateral lung. Due to the very low burden of progressive disease and the otherwise maintained systemic response, the patient underwent surgical resection of the lesion. NGS was assessed to evaluate the possible resistance mechanisms to BRAF-TKIs, and surprisingly no BRAF alterations emerged. Additional comprehensive genomic sequencing revealed a completely distinct molecular profile compared to the primary tumor, strongly supporting the hypothesis that the lesion represented a second primary lung malignancy rather than metastasis. Following resection, the patient maintained a partial response for at least 12 months after surgery, with an overall response duration of 34 months, underscoring the clinical value of local therapy in carefully selected patients with apparent oligoprogression [69]. Of note, regarding TRK fusion positive cancers, local therapy with TRK-TKI treatment beyond progression in case of solitary site progression or oligoprogression could be considered [60].

Several clinical trials are ongoing to assess the role of local ablative therapy in oncogene-addicted NSCLC during TKI treatment. Interestingly, the latest trials involve *other* oncogenic drivers (Table 1).

HALT is a phase II/III, multicenter, randomized controlled trial enrolling patients with advanced non-small-cell lung cancer harboring an actionable mutation responding to TKI therapy and with five or fewer sites of oligoprogressive disease. In this trial, the patients are randomized in a 2:1 ratio to receive SBRT or no SBRT, with all the patients continuing to receive background treatment with TKI therapy. Primary aim of this trial is to assess if SBRT on ≤3 sites of OPD, continuing TKI administration, improves the PFS rate compared with the treatment beyond progression alone [70]. In the SUPPRESS-NSCLC trial, 68 patients with oligoprogression to 1–5 extracranial lesions during systemic therapy will be randomized in a 1:1 ratio to receive standard of care (next-line systemic therapy, best supportive care, and continued current systemic line based on the physician’s choice) versus stereotactic ablative radiotherapy on all oligoprogressive lesions, while continuing their current systemic therapy. The patients must experience OPD during any line of Immune Checkpoint Inhibitor (ICI) or TKI, and patients with brain metastases are enrolled. As above, the primary endpoint is PFS. Lastly, NCT03808662 is an ongoing clinical trial which includes ROS1-rearranged OPD NSCLC, besides NSCLC with EGFR or ALK alterations and OPD triple-negative breast cancer. In this trial, OPD is defined with a cut-off of <5 progressing lesions. The aim of this trial is to determine if the addition of early SBRT to extra-cranial OPD could prolong PFS compared to the standard of care. Some translational research results may come from a trial investigating the role of TKI in a neoadjuvant setting, where surgical specimens of NSCLC already treated with target drugs could provide additional information about tumor clonality and potential resistance mechanisms. Along this vein, several trials with neoadjuvant TKIs are ongoing also in the context of NSCLC harboring *other* oncogenic drivers and could provide important resources to better understand the mechanisms underlying resistance and progression patterns in this setting (NAUTIKA-1 (NCT04302025) [71], Geometry-N trial [72], NCT05118854, and LIBRETTO-001 [73]).

## 5. Discussion

Despite the above-described data, the management of oligoprogression remains a significant clinical challenge, especially in relation to the *other* oncogenic drivers of NSCLC. Defining an appropriate therapeutic strategy requires careful evaluation of multiple factors, including timing, site of progression, tumor kinetics and the molecular context, all of which contribute to markedly heterogeneous clinical scenarios.

To date, no uniform definition of OPD has been established in the literature. OPD is generally described as the progression (increase in size and/or avidity defined as an increased radiotracer uptake on functional imaging such as FDG-PET) of 3–5 metastatic lesions during systemic therapy [74], considering not only the number, but also the anatomical site of OPD. OPD more frequently involves the CNS, the lungs, lymph nodes, and bones, whereas adrenal glands and the liver are less commonly affected [74].

From a mechanistic point of view, OPD may be related to the emergence of TKI-resistant clones, while the remaining tumor cell populations may retain sensitivity to systemic treatment. In this context, the use of local therapies allows for selective eradication of resistant lesions. This strategy is consistent with the concept of “clonal pruning”, whereby the targeted elimination of dominant resistant clones may temporarily preserve global sensitivity to targeted therapy, and consequently delay the initiation of next-line systemic treatment [75].

In this light, in patients with isolated CNS oligoprogression, continuation of an ongoing targeted therapy in combination with local brain-directed treatment may be an appropriate strategy. Alternatively, a switch to another targeted agent with established CNS activity may allow for postponement of local brain therapy [4]. This approach further highlights the need for an individualized balance between local disease control, preservation of neurocognitive function and maintenance of a systemic therapeutic benefit. Of note, Pisano et al. reported a case of a patient with ALK-rearranged metastatic lung adenocarcinoma who achieved prolonged disease control despite two intracranial oligoprogressions; they managed to achieve this while undergoing local therapy and continuing to take ALK-TKIs, maintaining a good quality of life [76].

The therapeutic evolution observed in EGFR-mutated and ALK-rearranged NSCLC has established and consolidated the paradigm of re-biopsy at progression. Building on this experience, the integration of tissue and liquid biopsies of progressing lesions has become the cornerstone of contemporary therapeutic decision making, with the rationale that similar principles may be reasonably extrapolated and applied to tumors harboring less common oncogenic drivers. Several studies have demonstrated that molecular characterization at the time of progression enables the identification of target-dependent resistance mechanisms as opposed to target-independent alterations or histological transformation, with substantial therapeutic implications. This is particularly relevant given the availability of novel targeted inhibitors addressing specific resistance alterations [77,78]. In EGFR mutant NSCLC, the resistance mechanisms include secondary mutations within the drug-binding site, most notably EGFR C797S, detected in roughly 15–20% of tumors progressing while the patient is on osimertinib, along with MET amplification or MET exon 14 skipping (approximately 15–20%), and histologic transformation to small-cell lung cancer (5–10%), frequently accompanied by RB1 and TP53 loss. Additional mechanisms include activation of alternative receptor tyrosine kinases (e.g., HER2, AXL, and IGF1R), epithelial–mesenchymal transition (EMT), and transcriptional reprogramming that reduces oncogene dependency [79,80]. In ALK-rearranged NSCLC, selective pressure from next-generation ALK inhibitors promotes kinase-domain resistance mutations such as G1202R; lorlatinib overcomes most single mutations, but selects for compound ALK mutations and bypass mechanisms. Compound ALK mutations, reactivation of downstream RAS–MAPK signaling, and bypass pathway engagement (including EGFR and KIT signaling) further contribute to therapeutic escape, often in conjunction with EMT-associated phenotypic changes [79,81]. Among the *other* alterations, KRAS G12C mutant NSCLC treated with covalent inhibitors such as sotorasib and adagrasib demonstrates clinically meaningful, but frequently transient responses. Resistance arises through secondary KRAS mutations that disrupt inhibitor binding (e.g., Y96D), activation of alternative RAS isoforms (NRAS and HRAS), MET amplification, and reactivation of MAPK or PI3K–AKT pathways [80]. ROS1 rearrangements, BRAFV600E mutations, RET fusions, and NTRK fusions display analogous resistance patterns characterized by gatekeeper or solvent-front mutations, oncogene amplification, pathway redundancy, and adaptive transcriptional remodeling [80,81].

However, in the context of OPD, significant limitations persist in the available evidence, including the lack of large, randomized trials, the heterogeneity of OPD definitions, as well as the limited prevalence of *other* oncogenic drivers. This underscores the need for prospective studies incorporating not only clinical endpoints, but also dynamic biomarkers (and possibly quality-of-life measures) to establish more definitive therapeutic algorithms.

## 6. Conclusions

*Other* oncogenic drivers are emerging in the landscape of oncogene-addicted NSCLC, allowing an increasing percentage of patients to receive a more tailored and less toxic therapy, with encouraging responses in clinical trials which are often maintained for a longer time than standard first-line chemotherapy. Sooner or later, with a pattern we already know in the setting of more common oncogenic drivers, resistance mechanisms occur, leading to disease progression. The optimal approach to OPD in the context of *other* oncogenic drivers has not been assessed yet, probably because of the lack of clinical data due to the very recent introduction of specific drugs and to the relative rarity of these alterations. Based on the available data on resistance mechanisms and borrowing from the experiences with major oncogenic drivers, we can assume that LAT may have a predominant role in this context, delaying the switch to second-line systemic therapy by controlling the single or few sites of progression. At present, only some data support this suggestion with scientific evidence, mainly coming from case reports. A systematic and multicentric collection of data from these patients is needed to provide more solid evidence. Moreover, with the increasing use of TKIs in the early stages, translational studies in clinical trials involving *other* oncogenic divers should be improved to collect specimens which could provide information on predictive biomarkers of resistance and relapse, allowing us to further select patients who require a closer follow-up or a more aggressive treatment.

## Figures and Tables

**Table 1:** Clinical trials exploring role of local ablative therapy (LAT) in oligoprogressive (OPD) NSCLC, including patients treated with TKIs for *other actionable* oncogenic drivers beyond EGFR and ALK.

Phase	Clinicaltrials.gov	Setting	N	Treatment	Primary Endpoints	Secondary Endpoints	Status	Results
II/III	NCT03256981 (HALT trial)	OPD in ≤3 sites after initial response to TKI (any actionable mutation allowed)	110	SBRT vs. no SBRT (2:1) + continuation of same TKI	PFS	Time to next line or to palliative care; OS; imaging patterns of progression; RT toxicity; QoL; resistant subclones on ctDNA; time to failure on next line	Unknown	NA
II	NCT04405401 (SUPPRESS-NSCLC)	OPD in 1–5 extracranial lesions ≤ 5 cm and involving ≤3 organs during ICI or TKI therapy	68	SABR + continuation of same systemic therapy vs. SoC (subsequent systemic therapy, BSC or treatment beyond progression)	PFS, OS	QoL, Grade > 3 toxicity, local control, time to next systemic therapy.	Recruiting	NA
II	NCT03808662 (PROMISE-004)	TNBC and NSCLC with OPD in ≤5 during systemic therapy, including EGFR, ALK or ROS1 TKI	106	SBRT + SoC vs. SoC	PFS	OS	Complete	NSCLC: mPFS 10 vs. 2.2 mo; (HR = 0.41; *p* = 0.0039)

*Legend*: N, number of patients; OPD, oligoprogressive disease; SBRT, Stereotactic Body Radiation Therapy; PFS, progression-free survival; OS, overall survival; QoL, quality of life; ctDNA, circulating tumor DNA; ICI, Immune Checkpoint Inhibitor; TKI, tyrosine kinase inhibitor; SABR, Stereotactic Ablative Radiotherapy; SoC, standard of care; BSC, best supportive care; TNBC, triple-negative breast cancer; NSCLC, non-small-cell lung cancer; NA, Not Available.

## Data Availability

No new data were created or analyzed in this study. Data sharing is not applicable to this article.
